# Explanation of somatic symptoms by mental health and personality traits: application of Bayesian regularized quantile regression in a large population study

**DOI:** 10.1186/s12888-019-2189-1

**Published:** 2019-07-03

**Authors:** Shayan Mostafaei, Kourosh Kabir, Anoshirvan Kazemnejad, Awat Feizi, Marjan Mansourian, Ammar Hassanzadeh Keshteli, Hamid Afshar, Saeed Masoud Arzaghi, Saeid Rasekhi Dehkordi, Peyman Adibi, Fataneh Ghadirian

**Affiliations:** 1 Department of Community Medicine, Faculty of Medicine, Kermanshah University of Medical Sciences, Kermanshah, Iran; 2 Department of Community Medicine, Faculty of Medicine, Non-communicable Diseases Research Center, Alborz University of Medical Sciences, Karaj, Iran; 3 Department of Biostatistics, Faculty of Medical Sciences, Tarbiat Modares University, Tehran, Iran; 4 Department of Epidemiology and Biostatistics, School of Public Health, Isfahan University of Medical Sciences, Isfahan, Iran; 5 Integrative Functional Gastroenterology Research Center, Isfahan University of Medical Sciences, Isfahan, Iran; 6 Department of Medicine, University of Alberta, Edmonton, AB Canada; 7 Psychosomatic Research Center, Isfahan University of Medical Sciences, Isfahan, Iran; 8 Elderly Health Research Center, Endocrinology and Metabolism Population Sciences Institute, Tehran University of Medical Sciences, Tehran, Iran; 9 Department of Psychiatric Nursing, School of Nursing and Midwifery, Tehran University of Medical Sciences, Tehran, Iran; 10 Epidemiology and Biostatistics Unit, Rheumatology Research Center, Tehran University of Medical Sciences, Tehran, Iran; 11 International Network of Integrated Nursing (INICN), Universal Scientific Education and Research (USERN), Tehran, Iran

**Keywords:** Somatic symptom, Mental health, Personality traits, Bayesian regularized quantile regression

## Abstract

**Background:**

Somatic syndrome is one of the remarkably prevalent issues in primary health care and subspecialty settings. We aimed to elucidate multidimensional associations between somatic symptoms with major mental problems and personality traits in the framework of the quantile regression model with a Bayesian approach.

**Methods:**

A total of 4763 employees at Isfahan University of Medical Sciences and Health Services in Isfahan province, Iran, filled out four validated questionnaires including Hospital Anxiety and Depression Scale (HADS), NEO Questionnaire, General Health Questionnaire (GHQ) and PHQ-15 for somatic symptom severity. In addition, Functional Gastrointestinal Disorders (FGIDs) were determined using Rome IV criteria. Exploratory Factor Analysis (EFA) and Bayesian regularized quantile regression with adaptive LASSO penalization were applied for reduced dimension of somatic symptoms and variable selection and parameter estimation, respectively.

**Results:**

The 25 major somatic symptoms were grouped into four factors including general, upper gastrointestinal, lower gastrointestinal and respiratory by EFA. Stress, depression, and anxiety had significant effects on all of the four extracted factors. The effect of anxiety in each four extracted factors was more than stress and depression. Neuroticism and agreeableness had significant effects on all of the four extracted factors, generally (*p* < 0.05).

**Conclusions:**

Given the high prevalence of somatic symptoms and psychosomatic complaints in correlation with the diverse range of mental co-morbidities, developing more detailed diagnostic tools and methods is crucial; nonetheless, it seems that providing better interdisciplinary approaches in general medical practice is groundwork.

## Background

Somatic syndrome is one of the remarkably prevalent issues in primary health care and subspecialty settings [1]. There is a comprehensive list of symptoms such as pain, breathlessness, palpitations, numbness, and gastroenterological problems with no definite affinity to a given medical specialty [2]. One-third of the symptoms remain medically unexplained after doing the comprehensive medical tests [1]. Although there are different reports on the prevalence rate of somatization syndrome, it seems that 50% of the patients in primary health setting have no detectable organ dysfunction [3]. Notably, there has been a 20–50% increase in using care services and costs of outpatients, a 30% increase in admission rates, and higher prevalence of abdominal surgeries [4, 5]. The majority of previous studies emphasize that those with somatization symptoms have higher levels of disability, psychiatric morbidity, and state benefits are less likely to attend work [6–8]. Somatic symptoms are prevalent in people with some kinds of psychiatric disorders. For example, about 48% of both bipolar and unipolar depressions show higher somatic symptoms than general population [9].

There is abundant information on inter-relationships between physical and mental health in psychosomatic conditions. According to these data, there has to be either an organ dysfunction or a psychological explanation for such symptoms [10]. Some findings revealed that 66% of employees with somatization syndrome attributed their symptoms to psychological or both psychological and physical factors [5]. Psychological distress is a known mediator of somatization. Using meta-analysis, a Cochrane review in 2014 showed that psychological therapy delivered in primary or secondary care (hospital and outpatient settings, respectively), resulted in less severe symptoms at the end of the treatment [11].

The up-to-date findings show that the prevalence of depression and anxiety in somatization is 4–6 times higher than in general population [5]. Stress, depression and anxiety are psychological distresses accompanied by cognitive-affective disturbances. From a neuroscience perspective, it is believed that cognitive-affective science provides important additional insights into the neurocircuitry of somato-sensory amplification. It has been stated that neural correlates of cognitive-affective amplifiers are integrated into a neurocircuit framework for somatosensory processing. The cognitive-affective disturbances including appraisal (negative anticipation), attentional bias, pain catastrophizing, alexithymia, and negative emotion have amplifying effects on visceral-somatic processing [1].

In addition to cognitive-affective amplifiers, one of the most important predictors of psychological distress in somatization are personality traits. It has been revealed that 52.6% of the subjects with somatization and psychological distress have higher levels of neuroticism and lower extraversion [12]. A hypothesis states that one of the most clinically noticeable problems of the patient with somatization is personality dysfunction. The estimated comorbidity of somatization and personality malfunction varies from 48 to 72% [13].

Although some studies have revealed the relational effects of somatization, mental health and personality traits, a few explain the somatic symptoms by mental health and personality traits in a given population. Lack of studies clarifying our understanding of somatization specifically with a psychological approach reveals a knowledge gap in this area. A meta-ethnography on qualitative studies states that the general practitioners have problem with somatization syndrome since the epistemological incongruence have overlaps with usual disease models [14]. Although the latest approaches have focused on classification of somatic symptoms based on symptom grouping to understand correctly what patients bring to doctors, the culture could have major effects on symptom interpretation and clinical manifestations of somatic symptoms. Therefore, population studies in a specific culture could provide more evidence on normative symptoms in non-clinical groups and help classifying the somatization symptoms. One of the most valued advantages of quantile regression is its model robustness in the sense that it makes no distributional assumption to the error term other than its quantile. Furthermore, quantile regression produced estimates that were unbiased and had smaller mean square errors than conventional regression models. Also, multiple associations of psychosomatic disorders with mental problems and the big five personality traits in the framework of the quantile regression model, especially with Bayesian approach, has not been studied yet. Finally, we applied Bayesian regularized quantile regression in order to assess the associations between stress, anxiety, depression, and big five personality traits with the severity of somatic symptoms in an Iranian population-based study.

## Methods

### Participants

The present study is a descriptive-analytical with a cross-sectional design carried out between April 2010 and May 2010. Ethical approval was obtained from the institutional review boards of Isfahan University of Medical Sciences and permission to recruit participants was provided by the participating health centers [15].

The population was consisted of 20,000 employees of Isfahan University of Medical Sciences and Health Services in Isfahan province. Among these 20,000 cases, 10,500 were not of academic staff and mainly had executive tasks in 50 different centers in Isfahan province, consisting of hospitals, campuses and health centers. In order to increase the rate of responses and participation as well as accuracy of the information collected, questionnaires were distributed in two phases with short intervals (3–4 weeks). Response rate was 86.1 and 64.64 percentages in the first and second phases, respectively. Finally, after matching returned questionnaires in phase 2 with their equivalents in phase 1, we reached 4763 questionnaires.

### Measures

Data was obtained from our four questionnaires including Hospital Anxiety and Depression Scale (HADS), NEO Questionnaire, General Health Questionnaire (GHQ), and PHQ-15. In addition, Functional Gastrointestinal Disorders (FGIDs) were diagnosed by Rome IV criteria. Severities of somatic symptoms were assessed by PHQ-15 and Rome IV criteria. Participants completed the questionnaires with information regarding age, gender, marital status, level of education, BMI, level of physical activity and Level of perceived support as confounding variables.

#### Anxiety and depression

Anxiety and depression were assessed with 14-item Hospital Anxiety and Depression Scale (HADS) Questionnaire. Hospital Anxiety and Depression Scale is commonly used by doctors to determine the levels of anxiety and depression. The HADS is a 14-item scale generating ordinal data among which seven relate to anxiety and seven to depression. Each item on the questionnaire is scored from 0 to 3 meaning that the participants could score between 0 and 21 for either anxiety or depression [16]. For anxiety (HADS-A), this gave a specificity of 0.78 and a sensitivity of 0.9. For depression (HADS-D), this gave a specificity of 0.79 and a sensitivity of 0.83 [17].

#### Personality traits

The big five personality traits were assessed with 60-item (12 items per domain) NEO Questionnaire. The version we used is the shortened format of the original version containing 240 items [18]. This shortened version of NEO PI-R is called NEO Five-Factor Inventory (NEO-FFI) designed to take 10–15 rather than 45–60 min to administer. The five personality traits of the Five-Factor Model (FFM) are Neuroticism (N) including emotional instability and self-consciousness, anxiety, angry hostility, depression, impulsivity, vulnerability; Extraversion (E) consists of sociability, assertiveness and social interaction, warmth, gregariousness, activity, excitement seeking, positive emotions; Openness (O): cognitive disposition to creativity and aesthetics, fantasy, feelings and values; Agreeableness (A): tendency towards being sympathetic, trusting and altruistic, straightforwardness, compliance and modesty; and Conscientiousness (C): tendency towards dutifulness, order, achievement striving, self-discipline, deliberation and competence [19]. For the NEO-FFI internal consistencies were reported: Neuroticism = 0.85, Extraversion = 0.80, Openness = 0.68, Agreeableness = 0.75 and Conscientiousness = 0.83. Overall, the results of these studies have been shown acceptable validity and reliability of NEO among those populations [20].

#### Stress

Stress was evaluated using the 12-item General Health Questionnaire (GHQ). The General Health Questionnaire is a method of quantifying the risk of developing psychiatric disorders or to screen instrument of identifying psychological distress among adults in primary care settings. The original version was comprised of 60 items but the 12-item version is mostly used in epidemiological studies [21]. Cronbach alpha coefficient for the GHQ is ranged between 0.82 to 0.86. The instrument is considered reliable and has been translated into 38 different languages [22].

#### Somatization symptoms

Patient Health Questionnaire (PHQ-15) is an implement of assessing psychosomaticdisorders (or somatic symptoms) [23]. PHQ-15 is widely used as an open access screening instrument for somatization symptoms in different health care settings. Kocalevent R-D and et al. provided normative data with the PHQ-15 for different age groups and both genders. So, the main achievement of this study was to standardize the PHQ-15 with the provision of normative data from the general population. Moreover, evidence supports the reliability and validity of the PHQ-15 [24]. Functional gastrointestinal disorders (FGIDs) are diagnosed and classified using the Rome criteria. Rome IV is the newest classification and diagnostic criteria of FGIDs. Rome IV has a symptom-based and multicultural focus on FGDIs. According to Rome IV, the FGIDs are disorders of gut-brain interaction (DGBI) and defined as *“*a group of disorders classified by GI symptoms related to any combination of motility disturbances, visceral hypersensitivity, altered mucosal and immune function, gut microbiota, and/or central nervous system processing.”

### Statistical analyses

First, exploratory factor analysis was applied to make reliable factors of somatization symptoms. One of the most valued advantages of quantile regression is its model robustness in the sense that it makes no distributional assumption to the error term other than its quantile. Furthermore, quantile regression produced estimates that were unbiased and had smaller mean square errors. Since quantile regression does not normally assume a parametric likelihood for the conditional distributions, Bayesian methods uses a working likelihood. Because of heterogeneity amongst participants, non-normal distributions of somatization symptoms, advantages of Bayesian framework and importance of some the covariates, Bayesian adaptive lasso quantile regression was used along with Gibbs sampler algorithm as one of the Markov Chain Monte Carlo (MCMC) algorithms by “Brq” and “bayesQR” R packages for assessing the associations between mental problems (stress, anxiety, and depression) and the big five personality traits as predictor variables with each of the factors of somatization symptoms as dependent variables. In order to fit the model, R software version 3.5.1 was used with 20,000 iterations after discarding an additional 30,000 iterations as burn-in period. For estimating the parameter in each model, mean of estimated coefficient (β) was mentioned as the effect size with 95% Credible Interval (95% CI, Highest Posterior Density). Convergence of Markov chains was assessed by geweke diagnostic and Gelman-Rubin diagnostic statistically tests [25]. In order to make robust results, asymmetric Laplace distribution was applied to form the likelihood function and low-informative uniform distributions were applied as prior distributions. Consequently, asymmetric Laplace distribution and Gibbs sampler were found very effective strategies for modeling Bayesian quantile regression by sampling the parameters from their full conditional distributions [26–28]. The quantiles 0.25, 0.5 and 0.75, with thinning parameter = 1, were used for the Bayesian quantile regression model. All models were cross-validated using repeated 5-fold cross-validation. This cross-validation process was repeated five times, with each of the five subsamples used exactly once as the validation data and the other subsamples used as the training data. Then the five results could be averaged to produce a single estimation of the prediction error.

## Results

### Sample characteristics

The mean (SD) of the participants’ age was 36.58 (SD = 8.09, range = 19–70) years. A total of 56.1% were women and 81.2, 17.1 and 1.7% were married, single, and widowed/divorced, respectively. Educational status of the participants was as follows: 13.8% under high school diploma, 29.1% high school diploma and 57.1% academic degree. Also, 34.8% had more than 1 h physical activities and the mean (SD) of BMI was 25.7 (4.64).

### Somatization symptoms factors

After EFA, four factors were extracted from 25 somatization symptoms. The number of extracted factors has been identified by scree plot and Kaiser criterion, variance explained criterion and hypothesis testing and MAP technique (chi square statistic is 343.13, and *p*-value is 7.15e-20. In addition, all of eigenvalues are greater than one and all of absolute value of loading factors are greater than 0.3). Principal component analysis was used method for factor extraction. The results are shown of the extracted factors from psychosomatic items after Promax rotation by exploratory factor analysis (EFA). The Kaiser Meyer Olkin measure of sampling adequacy was 0.947 for our samples which shows the adequacy of samples for EFA. The *p*-value of Bartlett’s test of Sphericity was 0.001. Percentages of variance for each factor extracted of general, upper gastrointestinal, lower gastrointestinal, and respiratory was 15, 11, 11, and 8%, respectively (Table 1).

**Table 1:** Results of exploratory factor analysis

The extracted factors	Eigen value	Somatization symptoms (factor loading)
General	3.932	Dizziness (0.641), backache (0.638), joint pain (0.637), Headache (0.573), heartbeat (0.552), fatigue (0.536), Eye pain(0.533), Pain in the teeth and jaw (0.467), quake (0.43), Flushing (0.395), Throat and neck pain(0.326), Menstruation disorders (0.307), Dry mouth (0.305)
Upper gastrointestinal	2.914	Nausea(0.647), heartburn (0.565), Abdominal fullness (0.562), globus (0.543), Non heart chest pain (0.454)
Lower gastrointestinal	2.909	constipation (0.704), flatulence (0.676), stomach cramp (0.54), Anal pain (0.485)
Respiratory	1.967	rattle(0.725), roup (0.689), short winded(0.515)

### Somatization symptoms factors, stress, anxiety, depression, and personality traits adjusting to sex and age

More information on the four extracted factors of anxiety, depression, stress and big five personality traits are shown in Table 2.

**Table 2:** Descriptive statistics of four extracted factors of the 25-somatization symptoms, anxiety, depression, stress and big five personality traits based on the different Departments of Isfahan University of Medical Sciences and Health Services (IUMS), Iran (the SEPAHAN project), stratify on the sex and age (*n* = 4763)

Variables	Sex			Age		
	Men	Women	*P*-value	age < 40 yrs.	age > 40 yrs.	*P*-value
	Mean (SD)	Mean (SD)		Mean (SD)	Mean (SD)	
Psychosomatic Disorders						
General	9.1 (2.93)	10.8 (2.55)	< 0.001^*^	10.7 (2.70)	10.9 (2.76)	0.028
Upper gastrointestinal	3.6 (1.05)	3.9 (1.19)	< 0.001^*^	3.8 (1.18)	3.7 (1.16)	0.032
Lower gastrointestinal	3.5 (1.35)	4.0 (1.15)	< 0.001^*^	3.8 (1.27)	3.8 (1.23)	0.58
Respiratory	2.4 (0.72)	2.4 (0.71)	0.242	2.3 (0.71)	2.4 (0.71)	< 0.001
Mental Disorders						
Stress	0.2 (0.17)	0.3 (0.17)	< 0.001^*^	0.3 (0.17)	0.2 (0.18)	0.002
Anxiety	0.1 (0.12)	0.2 (0.12)	< 0.001^*^	0.2 (0.13	0.1 (0.11)	0.001
Depression	0.2 (0.21)	0.3 (0.22)	< 0.001^*^	0.3 (0.19)	0.3 (0.23)	0.275
Personality Traits						
Neuroticism	17.6 (6.87)	19.7 (8.76)	< 0.001^*^	19.3 (7.80)	17.9 (7.33)	< 0.001
Extraversion	29.9 (6.85)	28.4 (7.15)	< 0.001^*^	10.7 (6.10)	10.9 (8.23)	0.233
Agreeableness	23.7 (5.36)	24.3 (5.15)	< 0.001^*^	24.6 (4.91)	23.2 (5.45)	< 0.001
Openness	30.3 (6.96)	31.7 (6.53)	< 0.001^*^	31.5 (6.42)	30.8 (6.86)	0.001
Conscientiousness	36.0 (7.18)	36.4 (7.38)	0.056	36.6 (8.23)	36.1 (6.55)	0.09

* indicated statistical significant at level of 0.05

In Tables 3, 4, 5 and 6, the posterior mean and 95% equal-tail credible intervals (95% CI) from Bayesian adaptive LASSO quantile regression with adjusting confounding variables (such as age, gender, marital status, level of education, BMI, level of physical activity and Level of perceived support) are displayed in three quantiles.

**Table 3:** Results of Bayesian adaptive LASSO quantile regression for general somatization symptoms with adjusting confounding variables (e.g. sex and age)

Response variable	Exploratory variables	Quartile 1 (0.25) Coefficient (%95 CI)	Quartile 2 (0.5) Coefficient (%95 CI)	Quartile 3 (0.75) Coefficient (%95 CI)
General	Stress	0.63 (0.55, 0.70)	0.55 (0.49, 0.60)	0.61 (0.55, 0.68)
	Anxiety	1.47 (1.3, 1.55)	1.66 (1.59, 1.72)	1.96 (1.87, 2.05)
	Depression	0.95 (0.87, 1.03)	1.17 (1.10, 1.25)	1.05 (0.99, 1.11)
	Neuroticism	0.03 (0.02, 0.03)	0.04 (0.04, 0.04)	0.04 (0.04, 0.05)
	Extraversion	−0.04 (−0.04,−0.03)	− 0.03 (− 0.03, − 0.02)	-0.03 (− 0.03, − 0.02)
	Agreeableness	−0.02 (− 0.03, − 0.02)	−0.04 (− 0.04, − 0.03)	−0.05 (− 0.06, − 0.05)
	Openness	−0.02 (− 0.02,− 0.01)	0.00 (− 0.01, 0.00)	0.02 (0.01, 0.02)
	Conscientiousness	0.05 (0.05, 0.06)	0.03 (0.02, 0.03)	0.03 (0.02, 0.03)

**Table 4:** Results of Bayesian adaptive LASSO quantile regression for upper gastrointestinal somatization symptoms with adjusting confounding variables (e.g. sex and age)

Response variable	Exploratory variables	Quartile 1 (0.25) Coefficient (%95 CI)	Quartile 2 (0.5) Coefficient (%95 CI)	Quartile 3 (0.75) Coefficient (%95 CI)
Upper gastrointestinal	Stress	0.35 (0.24, 0.42)	0.23 (0.14, 0.30)	0.09 (0.05, 0.14)
	Anxiety	0.58 (0.53, 0.64)	0.73 (0.66, 0.78)	0.83 (0.76, 0.89)
	Depression	0.06 (0.02, 0.11)	0.29 (0.24, 0.34)	0.67 (0.63, 0.71)
	Neuroticism	0.01 (0.00, 0.01)	0.01 (0.01, 0.02)	0.03 (0.02, 0.03)
	Extraversion	0.00 (0.00, 0.00)	−0.01 (− 0.02, 0.00)	0.01 (0.00, 0.01)
	Agreeableness	0.00 (0.00, 0.00)	-0.01 (−0.02,0.00)	−0.02 (− 0.02, − 0.01)
	Openness	0.00 (− 0.01,0.00)	−0.01 (− 0.02, − 0.01)	−0.02 (− 0.02, − 0.01)
	Conscientiousness	0.01 (0.01, 0.02)	0.01 (0.00, 0.07)	0.01 (0.00, 0.01)

**Table 5:** Results of Bayesian adaptive LASSO quantile regression for lower gastrointestinal somatization symptoms with adjusting confounding variables (e.g. sex and age)

Response variable	Exploratory variables	Quartile 1 (0.25) Coefficient (%95 CI)	Quartile 2 (0.5) Coefficient (%95 CI)	Quartile 3 (0.75) Coefficient (%95 CI)
Lower gastrointestinal	Stress	0.15 (0.08, 0.22)	0.22 (0.18, 0.26)	0.22 (0.16, 0.27)
	Anxiety	0.40 (0.33, 0.47)	0.52 (0.46, 0.58)	0.64 (0.58, 0.71)
	Depression	0.24 (0.18, 0.30)	0.25 (0.21, 0.30)	0.34 (0.29, 0.39)
	Neuroticism	0.01 (0.01, 0.02)	0.01 (0.01, 0.01)	0.02 (0.02, 0.03)
	Extraversion	0.00 (− 0.01, 0.00)	−0.01 (− 0.01,0.00)	0.00 (0.00, 0.00)
	Agreeableness	0.01 (0.01, 0.02)	0.00 (−0.01, 0.00)	0.00 (−0.01,0.00)
	Openness	−0.02 (− 0.02, − 0.02)	−0.02 (− 0.03, − 0.02)	−0.01 (− 0.01,0.00)
	Conscientiousness	0.02 (0.01, 0.02)	0.03 (0.03, 0.03)	0.02 (0.02, 0.02)

**Table 6:** Results of Bayesian adaptive LASSO quantile regression for respiratory somatization symptoms with adjusting confounding variables (e.g. sex and age)

Response variable	Exploratory variables	Quartile 1 (0.25) Coefficient (%95 CI)	Quartile 2 (0.5) Coefficient (%95 CI)	Quartile 3 (0.75) Coefficient (%95 CI)
Respiratory	Stress	0.00 (0.00, 0.01)	0.10 (0.05, 0.14)	0.04 (0.00, 0.09)
	Anxiety	0.00 (0.00, 0.01)	0.12 (0.07, 0.17)	0.48 (0.42, 0.53)
	Depression	0.00 (0.00, 0.00)	0.04 (0.01, 0.09)	0.05 (0.01, 0.09)
	Neuroticism	0.00 (0.00, 0.00)	0.01 (0.00, 0.01)	0.01 (0.00, 0.01)
	Extraversion	0.00 (0.00, 0.00)	−0.01 (− 0.01,0.00)	0.00 (0.00, 0.01)
	Agreeableness	0.00 (0.00, 0.00)	−0.02 (− 0.03, − 0.02)	−0.01 (− 0.01, − 0.01)
	Openness	0.00 (0.00, 0.00)	0.01 (0.00, 0.01)	0.00 (0.00, 0.00)
	Conscientiousness	0.00 (0.00, 0.00)	0.04 (0.03, 0.04)	0.0 0.00, 0.01)

### Explaining general somatization symptoms

Stress, depression, anxiety, neuroticism and conscientiousness significantly increased the general somatization symptoms. Interestingly, anxiety and extraversion had the most increasing and decreasing effects on the general somatization symptoms, respectively (Table 3).

### Explaining upper gastrointestinal somatization symptoms

According to Table 4, anxiety, depression, stress, neuroticism and conscientiousness had significant increasing effects on the upper gastrointestinal somatization symptoms. However, openness and agreeableness had the most decreasing effects on the upper gastrointestinal somatization symptoms.

### Explaining lower gastrointestinal somatization symptoms

Results of lower gastrointestinal somatization symptoms showed that anxiety, depression, stress, conscientiousness and neuroticism had significant increasing effects on the lower gastrointestinal somatization symptoms, respectively. Only openness showed decreasing effect on the lower gastrointestinal somatization symptoms (Table 5).

### Explaining respiratory somatization symptoms

According to Table 6, anxiety, stress and depression had significant increasing effects on the respiratory somatization symptoms. However, the increasing effects of conscientiousness and neuroticism are not significant on the respiratory somatization symptoms. Only agreeableness had significant decreasing effect on the respiratory somatization symptoms.

## Discussion

The objective of the current study was to explain the somatization syndrome through mental health and personality traits in Iranian population. Firoozabadi et al. (2015) reported that major mode of expression of psychological distress in familial and interpersonal relationships in Iranian population is somatization [29]. The classification of somatic symptoms in primary care is difficult, yet very important. There is not a unique bodily symptom profile for somatization syndrome due to numerous overlapping diagnoses and syndrome labels. Rosendal et al. (2017) believed that despite the clinical uncertainty and mind-body dualistic nature of somatization, it is vital to reach a classification [30]. The findings showed that there are four main categories (or factors) for 25 somatic symptoms to explain somatization by mental health and personality traits; general, upper gastrointestinal, lower gastrointestinal and respiratory. General symptoms include dizziness, backache, joint pain, headache, heartbeat, fatigue, eye pain, pain in the teeth and jaw, quake, flushing, throat and neck pain, menstruation disorders, dry mouth have higher percentage of variance. Studying to identify the profiles of psychosomatic disorders in an Iranian adult population, Shabbeh and et al. (2016) stated that four categories of psychosomatic disorders including mental, gastrointestinal, respiratory, and general symptoms were extracted from psychosomatic symptoms with 42.02% of the total variance [31]. Eliasen et al. (2017) reported in a latent class analysis in a Danish population-based health survey that the most common somatic symptoms included 6 categories: musculoskeletal (three symptoms), gastrointestinal (three symptoms), cardiopulmonary (four symptoms), general (four symptoms), urinary tract (two symptoms), and other symptoms (three symptoms) [32]. It seems that varied categories of bodily symptom profiles could be better explained by cultural differences.

Stress, depression and anxiety have significant increasing effects on all of the three quantiles in each of the extracted factors. However, these effects were less detected in the respiratory system than the other extracted factors. The effect of anxiety in each extracted factor was more than stress and depression. The greater impact of anxiety on somatic symptoms we found is not in agreement with some studies emphasizing on depression [33]. Liao et al. (2017) stated that the health anxiety is the most common feature of diagnostic criteria of subsyndromal psychosomatic conditions [34]. Also, the age and employment status difference of the participants in the current study may explain this discordance.

The result of the current large population-based study showed interesting great impact of personality dimensions on psychosomatic symptoms. According to the findings, neuroticism and agreeableness had great impacts on somatic symptoms; which in the case of neuroticism dimension is comparable to other large scale studies [35, 36]. Neuroticism and Agreeableness showed increasing effects on all the four extracted factors. A significant relation was also found between agreeableness and different somatic symptoms; consistent with self-regulation effects on health behavior and health promoting behavior [37, 38]. Atari and Yaghobirad (2016) assessed the big five personality dimensions and mental health in 257 Iranian general populations. Their study showed that basic personality traits are predictors of alexithymia and considered the mediating variables contributing to mental health. They concluded that the big five dimensions of personality predispose individuals to alexithymia and high neuroticism, low extraversion, and low conscientiousness may predict higher scores of alexithymia and then more somatic symptoms [39].

There are potential limitations to this study. Although, the major mode of expression of psychological distress among Iranian population is somatization, culturally, Iranians report the psychological distresses lessened compared to their actual condition. Also, not reporting the subthreshold somatic symptoms in Rome IV limits the classification possibility. This normative population study focused on a specific population with a young mean age and work environment that could potentially limit the study generalization. Additionally, the results of the advanced statistical model were complex and compose its own limitations of use in clinical practice.

This study brings up some interesting questions for further research. It seems necessary to find out how many categories (factors) we would have in other Iranian communities. Also, we could consider the neuroticism, agreeableness, and anxiety as extracted factors in different population studies. Given high prevalence of somatic symptoms and psychosomatic complaints in correlation with diverse range of mental co-morbidities, developing more detailed diagnostic tools and methods is crucial; nonetheless, it seems that providing better interdisciplinary approaches in general medical practice is groundwork. In addition, adopting appropriate psychiatric assessments and suitable psychotherapies could be effective to manage somatic symptoms and decrease the burden of illness.

## Conclusions

This normative survey showed the prevalence of normalcy of somatic symptoms at 4 factors between 8 and 15%. The findings suggest an exploratory model consisting of four somatic symptoms categories considering anxiety in mental health and neuroticism and agreeableness in personality traits at non-clinical Iranian population. The current fragmented approach to functional somatic symptoms due to the various syndrome diagnoses is an obstacle faced in research interfering with an effective care. Although it is known that the somatic symptoms have clearly distinct disease entities, it is believed that they rather represent a common phenomenon with different subtypes. Therefore, there is a strong will for developing explanations, which permit the biological and the psychosocial causes to co-exist in a wide range of symptoms and settings.

## Data Availability

The datasets used and/or analyzed during the current study are available from the corresponding author on reasonable request.

## Abbreviations

BMI: Body mass index
CI: Credible interval
DGBI: Disorders of gut-brain interaction
EFA: Exploratory factor analysis
FFM: Five-factor model
FGIDs: Functional gastrointestinal disorders
GHQ: General health questionnaire
HADS: Hospital anxiety and depression scale
IUMS: Isfahan university of medical sciences
MCMC: Markov chain monte carlo
PHQ: Patient health questionnaire
SD: Standard deviation

## References

[1] Perez DL, Barsky AJ, Vago DR, Baslet G, Silbersweig DA (2015). A neural circuit framework for somatosensory amplification in somatoform disorders. J Neuropsychiatry Clin Neurosci.

[2] Eriksen TE, Risør MB (2014). What is called symptom?. Med Health Care Philos.

[3] Isaac ML, Paauw DS (2014). Medically unexplained symptoms. Med Clin.

[4] Aziz I, Palsson OS, Törnblom H, Sperber AD, Whitehead WE, Simrén M (2018). The prevalence and impact of overlapping Rome IV-diagnosed functional gastrointestinal disorders on somatization, quality of life, and healthcare utilization: a cross-sectional general population study in three countries. Am J Gastroenterol.

[5] Nimmo SB. Medically unexplained symptoms. Occup Med. 2015;65(2):92–4. 10.1093/occmed/kqv004 25694487

[6] Poulsen OM, Persson R, Kristiansen J, Andersen LL, Villadsen E, Ørbæk P (2013). Distribution of subjective health complaints, and their association with register based sickness absence in the Danish working population. Scand J Public Health.

[7] Tomenson B, Essau C, Jacobi F, Ladwig KH, Leiknes KA, Lieb R (2013). Total somatic symptom score as a predictor of health outcome in somatic symptom disorders. Br J Psychiatry.

[8] Roelen CA, Koopmans PC, Groothoff JW (2010). Subjective health complaints in relation to sickness absence. Work.

[9] Edgcomb JB, Tseng C-H, Kerner B (2016). Medically unexplained somatic symptoms and bipolar spectrum disorders: a systematic review and meta-analysis. J Affect Disord.

[10] Payne H (2015). The body speaks its mind: the BodyMind Approach® for patients with medically unexplained symptoms in primary care in England. Arts Psychother.

[11] olde Hartman TC, Rosendal M, Aamland A, van der Horst HE, Rosmalen JG, Burton CD (2017). What do guidelines and systematic reviews tell us about the management of medically unexplained symptoms in primary care?. BJGP Open.

[12] Menon V, Shanmuganathan B, Thamizh JS, Arun AB, Kuppili PP, Sarkar S (2018). Personality traits such as neuroticism and disability predict psychological distress in medically unexplained symptoms: a three-year experience from a single centre. Personal Ment Health.

[13] Van Dijk S, Hanssen D, Naarding P, Lucassen P, Comijs H, Voshaar RO (2016). Big five personality traits and medically unexplained symptoms in later life. Eur Psychiatry.

[14] Johansen M-L, Risor MB (2017). What is the problem with medically unexplained symptoms for GPs? A meta-synthesis of qualitative studies. Patient Educ Couns.

[15] Adibi P, Keshteli AH, Esmaillzadeh A, Afshar H, Roohafza H, Bagherian-Sararoudi R, Daghaghzadeh H, Soltanian N, Feinle-Bisset C, Boyce P, Talley NJ. The study on the epidemiology of psychological, alimentary health and nutrition (SEPAHAN): overview of methodology. J Res Med Sci. 2012;17(5):S292–8.

[16] Zigmond AS, Snaith RP (1983). The hospital anxiety and depression scale. Acta Psychiatr Scand.

[17] Bjelland I, Dahl AA, Haug TT, Neckelmann D (2002). The validity of the hospital anxiety and depression scale: an updated literature review. J Psychosom Res.

[18] Costa P, McCrae RR (2010). The NEO personality inventory: 3.

[19] McCrae RR, John OP (1992). An introduction to the five-factor model and its applications. J Pers.

[20] Sherry SB, Hewitt PL, Flett GL, Lee-Baggley DL, Hall PA (2007). Trait perfectionism and perfectionistic self-presentation in personality pathology. Personal Individ Differ.

[21] Goldberg D, Williams P (2000). General health questionnaire (GHQ).

[22] Quek KF, Low WY, Razack AH, Loh CS (2001). Reliability and validity of the general health questionnaire (GHQ-12) among urological patients: a Malaysian study. Psychiatry Clin Neurosci.

[23] Kroenke K, Spitzer RL, Swindle R (1998). A symptom checklist to screen for somatoform disorders in primary care. Psychosomatics.

[24] Kocalevent R-D, Hinz A, Brähler E (2013). Standardization of a screening instrument (PHQ-15) for somatization syndromes in the general population. BMC Psychiatry.

[25] Gelman A, Rubin DB (1992). Inference from iterative simulation using multiple sequences. Stat Sci.

[26] Yu K, Moyeed RA (2001). Bayesian quantile regression. Statist Probab Lett.

[27] Gilks WR, Richardson S, Spiegelhalter D. Markov chain Monte Carlo in practice. Chapman and Hall/CRC; 1995.

[28] Li Q, Xi R, Lin N (2010). Bayesian regularized quantile regression. Bayesian Anal.

[29] Firoozabadi Ali, Bellissimo Nick, Ghanizadeh Ahmad, Nesseri Abrisham Tanhatan (2015). Somatization as a Major Mode of Expression of Psychological Distress in Familial and Interpersonal Relationships Among Iranian Women. Women's Mental Health.

[30] Rosendal M, Hartman TCO, Aamland A, Van der Horst H, Lucassen P, Budtz-Lilly A (2017). “Medically unexplained” symptoms and symptom disorders in primary care: prognosis-based recognition and classification. BMC Fam Pract.

[31] Shabbeh Z, Feizi A, Afshar H, Hassanzade Kashtali A, Adibi P (2016). Identifying the profiles of psychosomatic disorders in an iranian adult population and their relation to psychological problems. J Mazandaran Univ Med Sci.

[32] Eliasen M, Jørgensen T, Schröder A, Dantoft TM, Fink P, Poulsen CH (2017). Somatic symptom profiles in the general population: a latent class analysis in a Danish population-based health survey. Clin Epidemiol.

[33] Bekhuis E, Boschloo L, Rosmalen JG, Schoevers RA (2015). Differential associations of specific depressive and anxiety disorders with somatic symptoms. J Psychosom Res.

[34] Liao S-C, Chen I-M, Tu C-Y, Hsu C-K, Ma H-M, Lee M-T (2017). Subsyndromal psychosomatic concepts and personality traits in community adults. Compr Psychiatry.

[35] den Boeft M, Twisk JW, Terluin B, Penninx BW, van Marwijk HW, Numans ME (2016). The association between medically unexplained physical symptoms and health care use over two years and the influence of depressive and anxiety disorders and personality traits: a longitudinal study. BMC Health Serv Res.

[36] Noyes R, Langbehn DR, Happel RL, Stout LR, Muller BA, Longley SL (2001). Personality dysfunction among somatizing patients. Psychosomatics.

[37] Sirois FM, Hirsch JK (2015). Big five traits, affect balance and health behaviors: a self-regulation resource perspective. Personal Individ Differ.

[38] DeYoung CG (2015). Cybernetic big five theory. J Res Pers.

[39] Atari M, Yaghoubirad M (2016). The big five personality dimensions and mental health: the mediating role of alexithymia. Asian J Psychiatr.

